# Infrastructure for genomic interactions: Bioconductor classes for Hi-C, ChIA-PET and related experiments

**DOI:** 10.12688/f1000research.8759.2

**Published:** 2016-06-28

**Authors:** Aaron T. L. Lun, Malcolm Perry, Elizabeth Ing-Simmons

**Affiliations:** 1 Cancer Research UK Cambridge Institute, University of Cambridge, Cambridge, UK; 2 MRC Clinical Sciences Centre, Faculty of Medicine, Imperial College London, London, UK

**Keywords:** Hi-C, ChIA-PET, infrastructure, data representation, genomic interactions

## Abstract

The study of genomic interactions has been greatly facilitated by techniques such as chromatin conformation capture with high-throughput sequencing (Hi-C). These genome-wide experiments generate large amounts of data that require careful analysis to obtain useful biological conclusions. However, development of the appropriate software tools is hindered by the lack of basic infrastructure to represent and manipulate genomic interaction data. Here, we present the *InteractionSet *package that provides classes to represent genomic interactions and store their associated experimental data, along with the methods required for low-level manipulation and processing of those classes. The *InteractionSet *package exploits existing infrastructure in the open-source Bioconductor project, while in turn being used by Bioconductor packages designed for higher-level analyses. For new packages, use of the functionality in *InteractionSet *will simplify development, allow access to more features and improve interoperability between packages.

## Introduction

Techniques such as chromatin conformation capture with high-throughput sequencing (Hi-C) ^1^ and chromatin interaction analysis with paired-end tags (ChIA-PET) ^2^ are increasingly being used to study the three-dimensional structure and organisation of the genome. Briefly, genomic DNA is fragmented and subjected to a ligation step during which DNA from interacting loci are ligated together. High-throughput paired-end sequencing of the ligation products will identify pairs of interacting genomic regions. The strength of each interaction can also be quantified from the number of read pairs connecting the two interacting regions. This information can be used to derive biological insights into the role of long-range interactions in transcriptional regulation as well as the general organization of the genome inside the nucleus.

 The analysis of Hi-C and ChIA-PET data is not a trivial task, and many software packages have been developed to facilitate this process. Several of these packages like *diffHic* ^3^ and *GenomicInteractions* ^4^ are part of the open-source Bioconductor project, which aims to provide accessible tools for analyzing high-throughput genomic data with the R programming language. One of the strengths of the Bioconductor project is the quality and quantity of shared infrastructure available to developers ^5^. Pre-defined S4 classes such as GenomicRanges and SummarizedExperiment can be used to represent various types of genomic data and information, easing the maintenance burden for developers while also improving interoperability between packages for users. However, this kind of common infrastructure does not yet exist for the genomic interaction field. Instead, each package contains its own custom classes, which increases code redundancy and development load while reducing interoperability.

Here, we describe the *InteractionSet* package that provides base S4 classes for representing and manipulating genomic interaction data. It contains the GInteractions class, to represent pairwise interactions; the InteractionSet class, to store the associated experimental data; and the ContactMatrix class, to represent interactions in a matrix format. This facilitates code reuse across Bioconductor packages involved in analyzing data from Hi-C, ChIA-PET and similar experiments.

## Overview of available classes

### The GInteractions class

**Figure 1.**
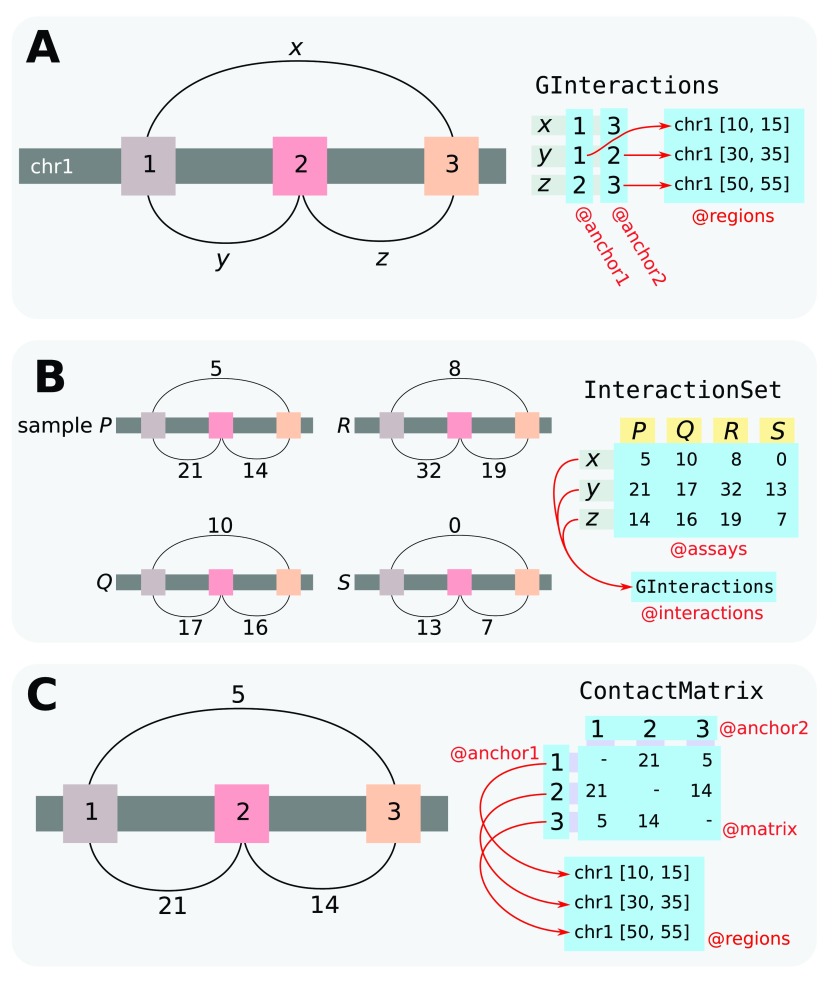
Overview of the classes in the *InteractionSet* package. Relevant slots of each class (i.e., data values stored in each object of the class) are labelled with a preceding “@”. ( **A**) The GInteractions class represents pairwise interactions between genomic regions by storing pairs of anchor indices that refer to coordinates in a GenomicRanges object. ( **B**) The InteractionSet class stores experimental data in an “assays” matrix where each row is an interaction and each column is a sample. Here, counts represent the number of read pairs mapped between each pair of interacting regions in each sample. ( **C**) The ContactMatrix class represents the interaction space as a matrix, where each cell represents an interaction between the corresponding row/column regions.

Each object of the GInteractions class is designed to represent interactions between pairs of “anchor” regions in the genome ( Figure 1A). It does so by storing pairs of anchor indices that point towards a reference set of genomic coordinates (specified as a GenomicRanges object). Each anchor index refers to a specific reference region, such that a pair of such indices represents a pairwise interaction between the corresponding regions. This design reduces memory usage as the reference coordinates need only be stored once, even if each region is involved in multiple interactions. Computational work is also reduced as calculations can be quickly applied across the small set of reference regions, and the results can be retrieved for each interaction based on the anchor indices. In addition, the GInteractions class inherits from the Vector class in Bioconductor’s *S4Vectors* package. This allows storage of metadata for each interaction (e.g., intensities, *p*-values) and for the entire object (e.g., experiment description).

### The InteractionSet class

The InteractionSet class is designed to store experimental data for each feature ( Figure 1B). It inherits from the SummarizedExperiment base class, where each object of the class stores any number of matrices of the same dimensions. Each row of each matrix corresponds to a pairwise genomic interaction (represented by a GInteractions object that is also stored within each InteractionSet object), while each column corresponds to an experimental sample. Each entry of the matrix then represents the observation for the corresponding interaction in the corresponding sample. Different matrices can be used to store different types of data, e.g., read counts, normalized intensities. The InteractionSet class also inherits a number of fields to store metadata for each interaction, for each sample, and for the entire object.

### The ContactMatrix class

The ContactMatrix class is designed to represent pairwise interactions in a matrix format ( Figure 1C). Each row and column of the matrix represents a genomic region, such that each cell of the matrix represents an interaction between the corresponding row/column regions. Experimental data for that interaction can be stored in the associated cell. This provides a direct representation of the “interaction space”, i.e., the two-dimensional space in which ( *x*, *y*) represents an interaction between *x* and *y*. Like the GInteractions class, the genomic coordinates are not stored directly – rather, the rows/columns have indices that point towards a reference set of coordinates, which reduces memory usage and computational work. The matrix representation itself uses classes in the *Matrix* package to provide support for both dense and sparse matrices. The latter may be more memory-efficient, particularly for sparse areas of the interaction space. ContactMatrix instances can also be easily converted to instances of existing matrix-based classes such as those in the *HiTC* package ^6^.

## Overview of available methods

**Figure 2.**
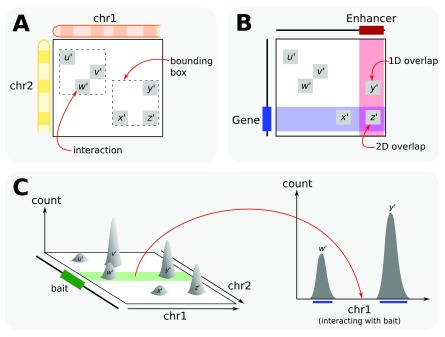
Schematic of several methods in the *InteractionSet* package. ( **A**) Minimum bounding boxes can be identified for groups of interactions using the boundingBox method. Here, *u*′, *v*′ and *w*′ belong in one group while *x*′, *y*′ and *z*′ belong in another. ( **B**) One- or two-dimensional overlaps can be identified between interactions and one or two genomic intervals, respectively, using the findOverlaps method. Here, *x*′ and *y*′ have one-dimensional overlaps with the gene and enhancer, respectively, while *z*′ has a two-dimensional overlap with the gene *and* the enhancer. ( **C**) An InteractionSet object contains data – in this case, read pair count data – for interactions in the two-dimensional interaction space. Given a bait region, a “cross-section” of the space can be extracted and converted into a RangedSummarizedExperiment object using the linearize method. This object holds count data for intervals on the linear genome (blue lines) where the count for each interval describes the strength of the interaction between that interval and the bait. This format effectively mimics that of 4C data.

The *InteractionSet* package provides a variety of methods for manipulating objects of each class. In addition to slot accessors and modifiers, methods are available to convert objects to different classes in the same package (e.g., GInteractions to ContactMatrix) or to base Bioconductor classes (e.g., GInteractions to GRangesList). The distance between anchor regions on the linear genome can be computed for each pairwise interaction, to use in fitting a distance-dependent trend ^1^ for diagnostics or normalization. The minimum bounding box in the interaction space can also be defined for a group of interactions ( Figure 2A) to summarize the location of that group.

The *InteractionSet* package supports one- or two-dimensional overlaps for its objects ( Figure 2B). A one-dimensional overlap is considered to be present between an interaction and a genomic interval if either anchor region of the interaction overlaps the interval. This can be used to identify interactions overlapping pre-defined regions of interest. A two-dimensional overlap is considered to be present between an interaction and two genomic intervals if one anchor region overlaps one interval and the other anchor region overlaps the other interval. This can be used to identify interactions linking two specific regions of interest, e.g., a gene and its enhancer. The same framework can be used to define two-dimensional overlaps between two interactions, based on whether the corresponding anchor regions overlap – this can be used to relate similar interactions in different GInteractions objects or across different experiments. More generally, interactions can be identified that link any two regions in a set of regions of interest. For example, given a set of genes, interactions between two genes can be identified; or given a set of genes and another set of enhancers, interactions linking any gene to any enhancer can be found.

Hi-C data in an InteractionSet object can also be converted into a 4C-like format ( Figure 2C). Firstly, a bait region is defined as some region of interest, e.g., a target gene or enhancer. All interactions in the InteractionSet object that have one-dimensional overlaps with the bait are identified. For each overlapping interaction, the anchor region that does *not* overlap with the bait is extracted and – along with the data associated with that interaction – used to construct a RangedSummarizedExperiment object. This process yields data for intervals on the linear genome, which is similar to the output of 4C experiments ^7^ that measure the intensity of interactions between the bait and all other regions. The “linearized” format may be preferable when a specific region can be defined as the bait, as intervals on the linear genome are easier to interpret than interactions in two-dimensional space.

## Implementation and operation details

All classes and methods in the *InteractionSet* package are implemented using the S4 object-orientated framework in R (version 3.3.0 or higher). Classes are exported to allow package developers to derive custom classes for their specific needs. Pre-existing Bioconductor classes and generics are used to provide a consistent interface for users. After loading the *InteractionSet* package into an R session, instances of each class can be constructed from existing data structures, either directly (e.g., GInteractions objects from GRanges via the GInteractions constructor, or from Pairs via the makeGInteractionsfromGRanges function; ContactMatrix objects from GRanges and Matrix via the ContactMatrix constructor) or in a hierarchical manner (e.g., InteractionSet objects from matrices and a GInteractions object via the InteractionSet constructor). The methods described above can then be applied to each instance of the class. While the *InteractionSet* package does not have functions to load data from file, it can be combined with the import function in the *rtracklayer* package ^8^ to construct class instances after importing data from a range of formats including BED and BEDPE. A similar strategy can be used to export data to file.

## Conclusions

The availability of common infrastructure is highly beneficial to software development by reducing redundancy and improving reliability, as more developers can check the same code; improving interoperability, as different packages use the same classes; and increasing the accessibility of useful features, which exist in a single package rather than being sequestered away in a variety of different packages. Here, we present the *InteractionSet* package that implements a number of classes and methods for representing, storing and manipulating genomic interaction data from Hi-C, ChIA-PET and related experiments. The package is fully integrated into the Bioconductor ecosystem, depending on a number of base packages to implement its classes (e.g., *S4Vectors*, *GenomicRanges*, *SummarizedExperiment*) while in turn being depended on by packages for higher-level analyses (e.g., *diffHic*, *GenomicInteractions*). Indeed, for any new packages, use of the features in *InteractionSet* will simplify development and improve interoperability with existing packages in the Bioconductor project. The *InteractionSet* package itself can be obtained for R version 3.3.0 at http://bioconductor.org/packages/InteractionSet.

## Software availability

Software and latest source code available from: http://bioconductor.org/packages/InteractionSet

Archived source code as at time of publication: http://dx.doi.org/10.5281/zenodo.51204 ^9^

License: GNU General Public License version 3.0
