# Supplementary material for: Infrastructure for genomic interactions: Bioconductor classes for Hi-C, ChIA-PET and related experiments
Source: F1000Res. 2016 Jun 28;5:950. Originally published 2016 May 20. [Version 2] doi: 10.12688/f1000research.8759.2 (PMC4890298; doi:10.12688/f1000research.8759.2)
Supplement: Peer review history

# Peer review: Referee response for version 1

**DOI:** 10.5256/f1000research.9426.r14093

**Published:** 2016-06-01

**Authors:** Nicolas Servant

**Affiliations:** 1 Institut Curie, Paris, France

The authors present the InteractionSet package, that eases the manipulation of chromosome conformation data within the BioConductor/R framework. The InteractionSet package was designed to store direct interactions between two genomic loci. It also proposed a ContactMatrix class allowing to store the interaction counts as a Matrix format. One important point is its ability to be generic allowing the manipulation of any type of interaction data, such as ChIA-PET, Hi-C or 4C data. This work provides an interesting base for package development in this field and should therefore be of great use to the community.

 In practice, the manuscript highlights the quality of the implementation and the optimization of this package. Dealing with Hi-C data can be challenging as the amount of data can be very large. Through this manuscript (Figure 1), it is clear that the authors propose an efficient strategy to manipulate such data.

 In addition, the package is well documented with a quick start guide (vignette) and the description of each function. The new classes are based on existing S4 classes and methods and should therefore be easy to use for users familiar with the intervals manipulation in R. The package is already used as a dependency of other packages such as GenomicInteractions and diffHiC. Finally, it is compatible with other existing BioConductor packages such as the HiTC package.

 Regarding the manuscript itself, it clearly describes what the InteractionSet does. It is well written and easy to read.

 I only have a few minor comments that I hope will help the authors to improve the manuscript and/or the package.

  

1. Storing direct interaction counts looks very interesting in practice. I'm just wondering how efficient is the GInteractions class in term of scalability and memory usage ? As an example, Rao *et al *recently generated Hi-C contact maps at a resolution of 5kb. This very high resolution dataset implies billion of Hi-C contacts. It would be interesting the know up to which resolution (or data throughput) the InteractionSet package is efficient, and/or which amount of RAM is require to deal with very large dataset.
2. The authors mentioned that the ContactMatrix class is compatible with matrix-based classes from the HiTC package. I therefore tried to convert a ContactMatrix object into a HTCexp object from HiTC (using the as() function) but It doesn't work. A note/example about that might be useful in the manual.
3. The package requires a recent version of R (>=3.3.0). It might be good to mention it somewhere.

I have read this submission. I believe that I have an appropriate level of expertise to confirm that it is of an acceptable scientific standard.

# Response

**Published:** 2016-06-02

**Authors:** Aaron Lun

**Affiliations:** Cancer Research UK Cambridge Research Institute, UK

Thanks for your comments Nicolas. Our responses are below:

***Storing direct interaction counts looks very interesting in practice. I'm just wondering how efficient is the GInteractions class in term of scalability and memory usage ? As an example, Rao et al recently generated Hi-C contact maps at a resolution of 5kb. This very high resolution dataset implies billion of Hi-C contacts. It would be interesting the know up to which resolution (or data throughput) the InteractionSet package is efficient, and/or which amount of RAM is require to deal with very large dataset.***

 In several Hi-C analyses that we have performed (50 kbp resolution, ~1 billion reads), the size of the InteractionSet object is around 100MB to 1GB. This is well within the capacity of modern desktop machines, let alone high performance computing (HPC) facilities. For smaller bin sizes, the quadratic increase in the potential number of bin pairs is mitigated by the fact that a greater number of those bin pairs will be empty. We only store non-empty interactions in GInteractions/InteractionSet objects, which avoids a quadratic increase in memory requirements. (In practice, further savings can be made by filtering to remove low-abundance interactions.)

 That said, for very large data sets with read coverage across the entire interaction space, the memory requirements will increase dramatically at higher resolutions. This can be mitigated to some extent by only operating on a single chromosome (or pair of chromosomes) at any given time. However, if this is not possible (e.g., the downstream analysis requires all interactions), then HPC resources and 64-bit R may be required to handle the resulting objects. We feel that such requirements are mostly unavoidable, as the generation of large data sets requires concomitant effort in the computational analysis.

***The authors mentioned that the ContactMatrix class is compatible with matrix-based classes from the HiTC package. I therefore tried to convert a ContactMatrix object into a HTCexp object from HiTC (using the as() function) but It doesn't work. A note/example about that might be useful in the manual.***

 Our concept of compatibility was based more on the class implementations and concepts, rather than through any explicit conversion. Specifically, both ContactMatrix and HTCexp use GRanges to represent the genomic coordinates of the row/column regions, and a Matrix class to represent store the intensity values across the interaction space. Thus, information extracted from an instance of a ContactMatrix class can directly be supplied to the HTCexp constructor:

*coords <- GRanges("chrA", IRanges(1:10, 1:10))*

*x2 <- ContactMatrix(Matrix(1:100, 10, 10), coords, coords) # dummy object*

*colnames(x2) <- rownames(x2) <- LETTERS[1:10] # dummy names*

*HTCexp(as.matrix(x2), anchors(x2, "column"), anchors(x2, "row"))*

 We are reluctant to implement this directly as an "as" method in the *InteractionSet* package, because we have tried to maintain a distinction between the low-level base classes in our package and the high-level analysis and visualization methods in other packages like *HiTC*, *diffHic*, *GenomicInteractions*, etc. Our hope is that developers who would like to use or be compatible with *InteractionSet* would write appropriate methods in their own packages to convert to/from classes as necessary.

**The package requires a recent version of R (>=3.3.0). It might be good to mention it somewhere.**

 Done.

# Peer review: Referee response for version 1

**DOI:** 10.5256/f1000research.9426.r13924

**Published:** 2016-05-25

**Authors:** Douglas Phanstiel

**Affiliations:** 1 Department of Genetics, Stanford University School of Medicine, Stanford, CA, USA

The authors describe an R package that allows users to store, organize, manipulate, and intersect pairwise chromosomal interactions. The package is written for use with HiC and ChIA-PET style data sets but could be used for a variety of pairwise interactions. While there exist a plethora of tools for the storage, manipulation, and analysis of single genomic elements, equivalent tools adapted for pairwise genomic interactions are limited and still greatly needed.

 The paper is well written, simple, and accurately describes the package itself. I have downloaded and tested the package and both download and usage went smoothly. It behaves similarly to some of the packages that it builds off of such as GenomicRanges. And those familiar with GenomicRanges will be at home when using InteractionSet. In addition to simply storing and organizing pairwise data, InteractionSet includes a lot of handy features that will be of great use to the community. These include simple functions that organize pairwise genomic interactions such as swapAnchors that assures that the first of the two paired regions is always on the lower numbered chromosome or upstream (with regard to Watson strand) and more complex functions such as findOverlaps that allows users to overlap sets of pairwise interactions in a variety of ways. 

 This package is very useful and powerful and provides a valuable resource to software developers and advanced users. The GenomicRanges-style organization of the data, that InteractionSet adopts, is often too complicated for casual R users to learn. In many cases simply reading files from BED, BEDPE, or sam format into data frames is easier and faster for simple tasks. However, developers will prefer this more standardized format for improved stability of their packages. And advanced users may prefer the standardized yet flexible approach to data organization and the powerful built in tools.

 Importantly, the package is accompanied by a detailed and clear online tutorial which clearly demonstrates how to use the classes and functions. In summary, this paper is succinct and clearly written and accurately describes an R package that will be of great use to the scientific community.

I have read this submission. I believe that I have an appropriate level of expertise to confirm that it is of an acceptable scientific standard.

# Response

**Published:** 2016-06-02

**Authors:** Aaron Lun

**Affiliations:** Cancer Research UK Cambridge Research Institute, UK

Thanks for your comments Douglas. We agree that the Bioconductor ecosystem of data classes can be somewhat daunting for new users. Nonetheless, we believe that the use of standard Bioconductor tools is the safest strategy for the majority of users (and obviously developers), given the number of "gotchas" in data processing, e.g., off-by-one issues in BED file loading.
